# Reply to Gücer, F.; Dünnebacke, J. Comment on “Hawez et al. Endometrial Intraepithelial Neoplasia, Concurrent Endometrial Cancer and Risk for Pelvic Sentinel Node Metastases. *Cancers* 2024, *16*, 4215”

**DOI:** 10.3390/cancers17081339

**Published:** 2025-04-16

**Authors:** Tabayi Hawez, Michele Bollino, Celine Lönnerfors, Jan Persson

**Affiliations:** 1 Division of Gynaecologic Oncologic Surgery, Department of Obstetrics and Gynaecology, Skåne University Hospital Lund, 22185 Lund, Sweden; 2 Department of Clinical Sciences, Obstetrics and Gynaecology, Faculty of Medicine, Lund University, 22185 Lund, Sweden

We thank you for your interest in our paper and would like to comment on your reflections [1].

The present study addresses the overall risk of endometrial cancer after histological examination following hysterectomy in women with a preoperative diagnosis of endometrial intraepithelial neoplasia (EIN) and also addresses morphologic endometrial factors associated with a final cancer diagnosis.

The FIGO 2009 staging system was followed in the present study as it was the staging system available at study commencement in 2019 and the basis for the treatment protocol. Although the FIGO 2023 staging system was published online in June 2023, the added histological and molecular features and the updated anatomical elements were not included in the Swedish national guidelines until June 18th 2024, a month after the enrollment of the last patient. Patients with either pelvic or para-aortic nodal involvement were thereby assessed according to the national guidelines and at the time correctly classified as stages IIIC1 and IIIC2.

The high proportion of ITCs in this study is coincidental and due to the limited sample size. A recent prospective study from our institution including 724 women with low-grade endometrioid endometrial cancer demonstrated a higher percentage of micro- and macrometastases compared to ITC [2].

The presence of ITC in a lymph node is definite proof of LVSI regardless of whether the primary tumor is diagnosed as LVSI positive. LVSI is regarded as an adverse prognostic factor in endometrial cancer. However, considerable interobserver variability exists, and we believe that LVSI and ITC should be interchangeable and that the logic in the FIGO 2023 classification on this issue is not watertight. For example, in our previous study, 58/155 (37.5%) of node-positive women were LVSI negative [2]. 

The prognostic significance of ITC has not been sufficiently studied. Micro- and macrometastases presumably originate from small tumor aggregates, potentially resulting in recurrence if left untreated. Furthermore, ITCs may be found in several SLNs (as in 3/6 “node-positive” women in the current study and in 35% of node-positive women in study [2], potentially representing a different risk profile [2]. In addition, a previous study found that small-volume pelvic nodal metastases were associated with a 16% risk of para-aortic nodal spread [3]. At the moment, the Swedish national guidelines recommend adjuvant oncological treatment in the case of ITCs in two or more SLNs.

Survival and recurrence data from adequately powered studies on women with low-grade endometrial cancer and small-volume SLN metastases including clearly defined LVSI data and biomarkers have yet to be published.

Considering the low risk of associated surgical trauma and lymphatic complications associated with SLN removal combined with a nodal involvement risk of up to 12.7%, the SLN procedure is suggested for women with presumed low-grade endometrial cancer [2]. This includes women with EIN as part of general endometrial thickening and/or women who obtained a preoperative diagnosis of EIN by endometrial biopsy, in whom the risk of endometrial cancer is 65%. In women with localized EIN in a polypoid lesion, SLN detection and removal can be omitted. 
